# Tracking gaze position from EEG: Exploring the possibility of an EEG‐based virtual eye‐tracker

**DOI:** 10.1002/brb3.3205

**Published:** 2023-09-18

**Authors:** Rui Sun, Andy S. K. Cheng, Cynthia Chan, Janet Hsiao, Adam J. Privitera, Junling Gao, Ching‐hang Fong, Ruoxi Ding, Akaysha C. Tang

**Affiliations:** ^1^ Department of Rehabilitation Sciences The Hong Kong Polytechnic University Hong Kong SAR China; ^2^ Department of Psychology The University of Hong Kong Hong Kong SAR China; ^3^ The Laboratory of Neuroscience for Education The University of Hong Kong Hong Kong SAR China; ^4^ Centre of Buddhism Studies The University of Hong Kong Hong Kong SAR China; ^5^ China Center for Health Development Studies Peking University Beijing China; ^6^ Neural Dialogue Shenzhen China; ^7^ Centre for Research and Development in Learning Nanyang Technological University Singapore

**Keywords:** BSS, eye movement, high‐density EEG, ICA, saccade, SOBI, smooth pursuit

## Abstract

**Introduction:**

Ocular artifact has long been viewed as an impediment to the interpretation of electroencephalogram (EEG) signals in basic and applied research. Today, the use of blind source separation (BSS) methods, including independent component analysis (ICA) and second‐order blind identification (SOBI), is considered an essential step in improving the quality of neural signals. Recently, we introduced a method consisting of SOBI and a discriminant and similarity (DANS)‐based identification method, capable of identifying and extracting eye movement–related components. These recovered components can be localized within ocular structures with a high goodness of fit (>95%). This raised the possibility that such EEG‐derived SOBI components may be used to build predictive models for tracking gaze position.

**Methods:**

As proof of this new concept, we designed an EEG‐based virtual eye‐tracker (EEG‐VET) for tracking eye movement from EEG alone. The EEG‐VET is composed of a SOBI algorithm for separating EEG signals into different components, a DANS algorithm for automatically identifying ocular components, and a linear model to transfer ocular components into gaze positions.

**Results:**

The prototype of EEG‐VET achieved an accuracy of 0.920° and precision of 1.510° of a visual angle in the best participant, whereas an average accuracy of 1.008° ± 0.357° and a precision of 2.348° ± 0.580° of a visual angle across all participants (*N* = 18).

**Conclusion:**

This work offers a novel approach that readily co‐registers eye movement and neural signals from a single‐EEG recording, thus increasing the ease of studying neural mechanisms underlying natural cognition in the context of free eye movement.

## Graphical abstract

We present the first prototype of an EEG‐based virtual eye‐tracker achieving an accuracy of 0.920° and precision of 1.510° of visual angle in a representative participant, with an average accuracy of 1.008 ± 0.357? and precision of 2.348 ± 0.580? of visual angle across all participants. This work offers a novel approach that readily co‐registers eye movement and neural signals from EEG recording.

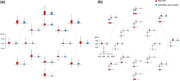

## 1 INTRODUCTION

Electroencephalography (EEG) and EEG‐based source imaging have been widely used in basic, clinical, educational, and commercial neuroscience research and applications (Cavanagh, 2018; Dikker et al., 2017; Donchin et al., 2000; He et al., 2018; Khushaba et al., 2013; Lau‐Zhu et al., 2019; Luck, 2014; McLoughlin et al., 2014; Niedermeyer & Lopes Da Silva, 2004; Privitera et al., 2022; Protzak & Gramann, 2018; Zink et al., 2016). Although advances have been made in the development of new electrodes for better signal quality (Casson, 2019) and ease of application (Kam et al., 2019; Lopez‐Gordo et al., 2014), the presence of large amplitude artifacts associated with eye movement continues to constrain the utility of EEG in the investigation of neural processes underlying natural cognition. To address this issue, typical EEG experimental paradigms require the fixation of the gaze at a specific location prior to and during stimulus presentation. Consequently, EEG‐based brain and behavior research is seldom conducted under the natural conditions of free eye movement, with few exceptions (Dimigen et al., 2011; Nikolaev et al., 2016).

Persistent efforts have been made over the past decades to identify, separate, and remove components associated with eye movements and other artifacts from EEG signal (Ranjan et al., 2021; Wallstrom et al., 2004). Blind source separation (BSS) methods, including independent component analysis (ICA) and second‐order blind identification (SOBI) (Bell & Sejnowski, 1995; Belouchrani et al., 1997), are typically used to separate and remove artifactual components from the original EEG in order to improve the quality of the neural signal (for reviews, see Tang 2010; Tang et al., 2011; Dimigen et al., 2011; Croft et al., 2000; Mannan et al., 2018; Jiang et al., 2019; Uriguen et al., 2015; Islam et al., 2016; Croft et al., 2005; Jung et al., 2000; Joyce et al., 2004; Bridwell et al., 2018). Today, the identification of ocular components from EEG is achieved through automatic classification or selection algorithms (Sun et al., 2021; Chaumon et al., 2015; Delorme et al., 2001; Dimigen et al., 2011; Joyce et al., 2004; Kierkels et al., 2007; Li et al., 2006; Mognon et al., 2011; Nolan et al., 2010; Plochl et al., 2012; Raduntz et al., 2015; Viola et al., 2009). In one example, Plochl et al. (2012) used eye movement data collected by an eye‐tracker to automatically identify and exclude five types of ocular artifact‐related components separated by ICA from EEG signal with a true positive success rate of 99%. Moreover, Many ICA selection algorithms, such as SASICA (Chaumon et al., 2015), FASTER (Nolan et al., 2010), and ADJUST (Mognon et al., 2011), have been developed to automatically identify different classes of artifacts (like blinks, vertical and horizontal eye movements, and generic discontinuities) with good success rates. Most previous works used approaches that treat the separated ocular and artifactual components as contaminations to be removed. They shared an implicit assumption that the extracted ocular signals are indeed ocular in origin. Recently, Castellanos and Makarov (2006) showed that ICA‐identified ocular components are actually contaminated by neural signals. Such contamination of artifact components by neural signals went unexamined, with few studies providing a systematic and quantitative measurement and statistical analysis of underlying sources of identified artifacts.

Recently, a novel SOBI–discriminant and similarity (DANS)‐based approach was developed to quantitatively validate extracted horizontal and vertical eye movement–related ocular artifact components and set an upper bound for any potential “contamination” of these components by remaining neural signals represented by the amount of unexplained variance (Sun et al., 2021). Spatially, these horizontal and vertical eye movement–related components, *H* and *V* components (*H* and *V* Comps), can be modeled as a pair of equivalent current dipoles with an ocular, nonneural origin. In the majority of participants studied, the neural contamination of SOBI‐identified ocular components was less than 5% for both *H* and *V* Comps. Further quantitative validation of the components’ ocular origin was provided by the systematic modulation of amplitudes of the *H* and *V* Comps as a function of saccade directions and distances, indicated by an effect size measure. Therefore, these components are more than mere artifacts but potentially contain useful signals indicating the gaze positions of individual participants. Furthermore, the systematic modulation of *H* and *V* Comps’ amplitude by gaze direction and distance was found not merely in group statistics but in statistics for each individual. These results raise the possibility that these eye movement–related components recovered from EEG data alone could be used to construct individual‐specific models for predicting gaze positions. If this could be achieved, one would be equipped with temporally synchronized neural signals related to cognitive processing and eye movement signals related to behavioral output, thus avoiding the additional demands and constraints associated with using a separate eye‐tracker and having to co‐register with the EEG. Building on this previous work (Sun et al., 2021), in an earlier feasibility study (Sun et al., 2020), we proposed a novel method for tracking eye movement using only the ocular component from EEG signal: an EEG‐based virtual eye‐tracker (VET). We believe this method can facilitate the study of neural mechanisms supporting visual perception and language functions in the natural context of free eye movement, like Egurtzegi et al. (2022).

Here, we describe our prototype of the EEG‐VET to show that it is possible to achieve a reasonable level of accuracy and precision. In this study, two kinds of eye movements tasks were used The first dot tracking trask, consisting of a sequence of directed saccadic eye moments to 8 directions and 2 distances to generate EEG data for estimating the parameters for the predictive models behind the EEG‐VET including determining accuracy and precision, whereas smooth pursuit eye movements were used to evaluate the performance of model prediction in terms of the root mean square error (RMSE) between the target and predicted gaze position. The results of this study strengthen our previous finding that SOBI–DANS identified ocular components capture signals coding for eye movement, and further demonstrate that these signals can be used to construct predictive models for tracking eye gaze without the need for an eye‐tracker. With further optimization, it is our hope that EEG‐VET will not only impact the study of neural mechanisms underlying natural cognition in the context of free eye movement but also provide a convenient way for tracking eye movement in EEG studies.

## 2 MATERIALS AND METHODS

### 2.1 Participants

Based on the sample size used in previous related works (Brooks et al., 2019; Niehorster et al., 2020), 25 healthy adults were initially included in the present study, 7 participants were excluded due to poor behavioral performance (fixation duration of 10% trials below than 300 ms, which follow the calibration procedure of commercialized eye‐trackers; *n* = 3), problems with eye‐tracking (missing trials >10%; *n* = 2), and EEG data quality (impedances of 1/3 electrodes >20 kΩ; *n* = 2). The final sample consisted of 18 participants (11 females) with a mean age of 20.71 years (SD = 1.83). Ethical approval (EA1801033) of this study was granted by the Human Research Ethics Committee of the University of Hong Kong, and written informed consent was obtained from each participant before the experiment.

### 2.2 Experimental tasks

**FIGURE 1:**
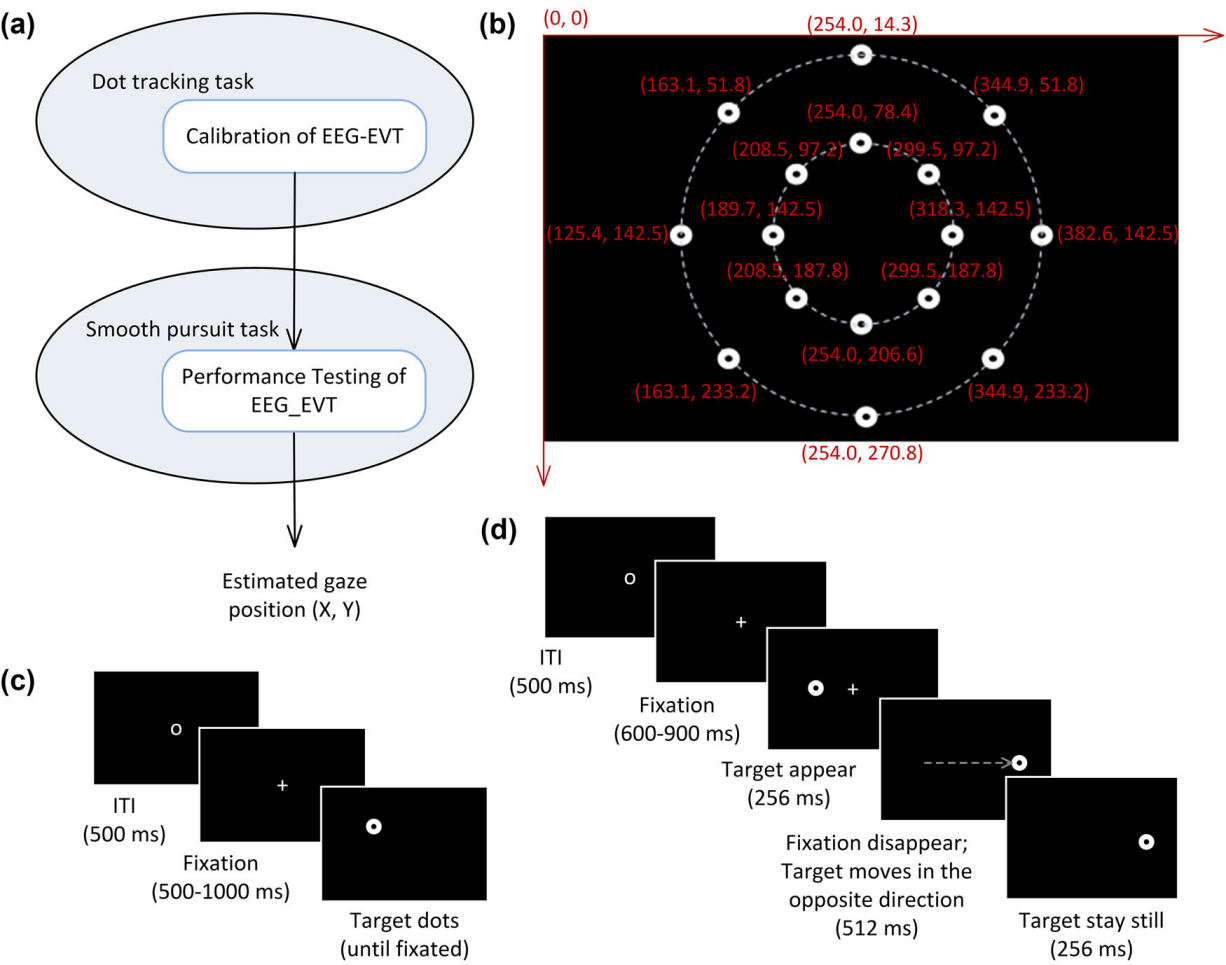
Methods: (a) study design flowchart; (b) the position of target dots in the dot tracking task; (c) dot tracking task; and (d) smooth pursuit task.

Tasks were administered using E‐prime 2.0 software (Psychology Software Tools). Participants were asked to sit in front of a computer screen in a quiet room, and their head position was stabilized by a chinrest. The chinrest was adjusted such that participants’ eye level was aligned with the screen center with a viewing distance of 600 mm. The experiment was composed of two tasks: dot calibration task and smooth pursuit task. The dot tracking task was used to estimate model parameters of the EEG‐VET, whereas the smooth pursuit task was used to test the performance (i.e., accuracy, precision, and RMSE) (Figure 1a).

#### 2.2.1 Dot tracking task

This task consisted of 32 directed eye movements from a central fixation point to one of 32 black dots presented on a white background, one at a time, at 16 possible locations, with 2 trials for each location (Figure 1b). Among the 16 possible locations, 8 locations were 12.2° of visual angle away from the screen center, and the other 8 locations were 6.1° of visual angle away from the screen center. These formed inner and outer rings of short versus long‐distance saccade target locations. In both conditions, the eight locations were evenly distributed among all possible directions from the screen center (0°, 45°, 90°, 135°, 180°, 225°, 270°, and 315° of visual angle). Each dot was a circle subtending about 0.6° of visual angle (or a diameter of 16 pixels). We define the upper left corner of the screen as the coordinate origin with the *X* axis extending to the right and the *Y* axis extending downward; the coordinates of the 16 target dots (given in mm) can be observed in Figure 1b. Participants were instructed to follow a sequence of dots presented at different locations in a random order, one at a time (Figure 1c). Prior to each trial, participants were told to focus on a fixation symbol “O” displayed in the center of the screen. A trial was initiated by the experimenter when the participant was fixated at the symbol “O.” The trial then began with a fixation cross “+” displayed at the center of the screen for a random duration between 500 and 1000 ms. Immediately after the offset of the fixation cross, a target dot appeared at one of the 16 possible locations randomly, to which the participant was instructed to move their eyes as fast and accurately as possible while avoiding blinks. Once a stable fixation on the target dot was detected by the eye‐tracker, the dot disappeared and participants were allowed to blink until they saw the fixation symbol “O” again. This task took approximately 3 min.

#### 2.2.2 Smooth pursuit task

This task consisted of 64 smooth pursuit eye movements with 8 trials for 4 speeds (1, 5, 9, and 19°/s) in 2 directions (left and right) (Figure 1d). Prior to each trial, participants looked at the center of the screen where a fixation symbol “O” was displayed for a minimum of 500 ms. A trial was initiated by the experimenter when the participant was fixated at the symbol “O.” The trial then began with a fixation cross “+” displayed at the center of the screen for a random duration between 600 and 900 ms. Participants were instructed to remain fixated until the target dot appeared. Immediately after the offset of the fixation cross “+,” a pursuit target dot appeared 2° of visual angle to the left (229, 142.5 mm) or right (275, 142.5 mm) of the fixation cross to prepare the participant to make a smooth pursuit (Braun et al., 2017). After 256 ms, the fixation cross disappeared, and the pursuit target dot began to move for 512 ms at one of the four possible speeds (1, 5, 9, or 19°/s) randomly in the opposite direction of the step. After stopping, the pursuit target dot remained stationary for 256 ms. This task took approximately 10–14 min.

### 2.3 Eye movement and EEG data acquisition and processing

Eye movement data were continuously collected at a sampling rate of 60 Hz using an SMI REDn eye‐tracker (iMotions), which was attached to a 17.5‐inch monitor with a screen resolution of 1280 × 720 pixels. Because the algorithm of the EEG‐VET does not involve the use of eye‐tracker data, these data were only used to exclude participants who missed >10% of task trials. Only data from the dominant eye were used for eye‐tracking data analysis. EEG data were recorded continuously along with eye‐tracking data using an eegoMylab system (ANT Neuro) with 64 electrodes placed according to the standard 10–20 system at a sampling rate of 500 Hz, with reference electrodes placed on the mastoids (the bones behind the ears) and electrode impedance kept below 20 kΩ. EEG data were notch‐filtered (50 Hz) and high‐pass filtered (0.1 Hz) before further processing. E‐prime software communicated with the EEG system via a parallel port.

### 2.4 Design of the EEG‐VET

**FIGURE 2:**
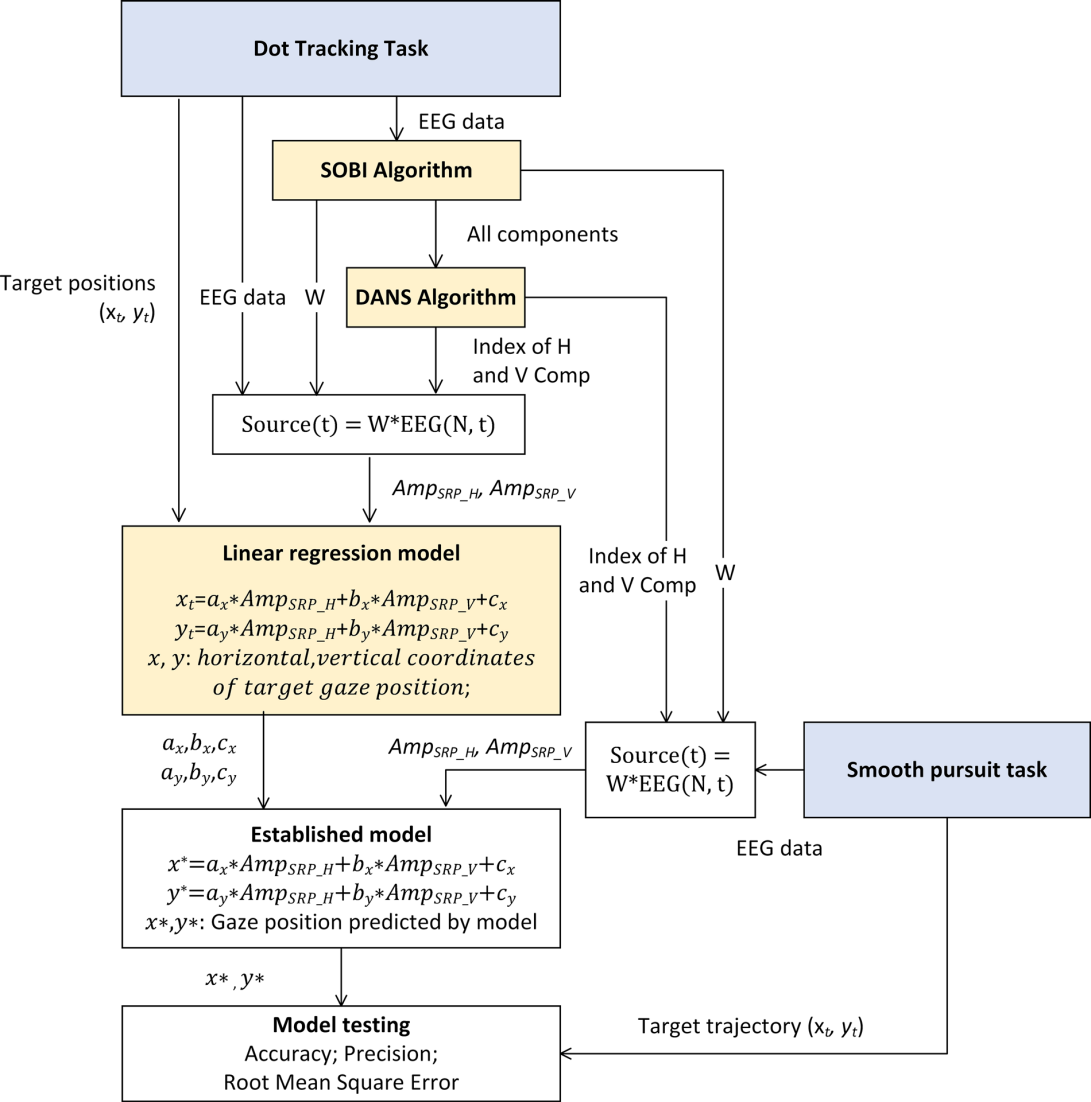
Flowchart of model calibration and performance testing of the electroencephalogram‐based virtual eye‐tracker (EEG‐VET). EEG‐VET mainly consists of three parts: second‐order blind identification (SOBI), discriminant and similarity (DANS), and linear regression analysis. It estimates the model parameters (*W* matrix, coefficients of independent variables, and intercepts) using data from the dot tracking task. The performance of EEG‐VET (accuracy, precision, and root mean square error [RMSE]) is assessed using data from both the dot tracking and smooth pursuit tasks. Note that each model is individual specific and does not require the pooling from group data.

Prior to use, EEG‐VET requires calibration to separate EEG signals into different components, identify ocular components from the recovered components (i.e., *H* and *V* Comps), and establish a linear regression model between ocular components and gaze position. The calibration algorithm includes an SOBI algorithm (Belouchrani et al., 1993, 1997) to decompose EEG signals, then the a DANS algorithm (Sun et al., 2021) to identify the horizontal and vertical ocular components, and finally a linear regression model (see flowchart in Figure 2) to map the identified ocular components to gaze positions.

#### 2.4.1 Decomposing EEG using SOBI algorithm

SOBI was applied to continuous EEG to decompose the 64‐channel data into 64 components, each of which corresponds to a recovered putative source contributing to the scalp‐recorded EEG signals. Detailed descriptions of SOBI's usage (Sutherland & Tang, 2006; Tang et al., 2002a; Tang et al., 2002b; Tang, Liu, et al., 2005; Tang, Sutherland and Wang et al., 2006), validation (Lio & Boulinguez, 2013; Tang, Sutherland, et al., 2005), and a review of SOBI usage (Tang, 2010; Tang et al., 2011; Urigüen & Garcia‐Zapirain, 2015) can be found elsewhere. Here we briefly introduce SOBI algorithm: Let *x*(*t*) represent the *n* continuous time series from the *n* EEG channels, where $x_{i}\left(t\right)$ corresponds to the *i*th EEG channel. Because various underlying sources are summed via volume conduction to give rise to the scalp EEG, each of the $x_{i}\left(t\right)$ is assumed to be an instantaneous linear mixture of *n* unknown components or sources $s_{i}\left(t\right)$, via an unknown *n* × *n* mixing matrix *A*, where x(t)=As(t). The putative sources ${\hat{s}}_{i}\left(t\right)$ are given by ${\hat{s}}_{i}\;\left(t\right)=\;Wx\left(t\right)$, where $W\;=A^{-1}$. SOBI finds the unmixing matrix *W* or mixing matrix *A* through an iterative process that minimizes the sum squared cross correlations between one recovered component at time *t* and another at time t+τ, across a set of time delays. The following set of delays, $\tau_{s}$ (in ms), was chosen to cover a reasonably wide interval without extending beyond the support of the autocorrelation function:

τ∈{1,2,3,4,5,6,7,8,9,10,12,14,16,18,20,25,30,35,40,45,50,55,60,65,70,75,80,85,90, 95,100,125,150,175,200,225,250,275,300,325,350}.

The calculation of mixing matrix *A* and source signals ${\hat{s}}_{i}\left(t\right)$ is described in Belouchrani et al. (1997).

#### 2.4.2 Identifying ocular components using DANS algorithm

Our previously validated DANS algorithm was applied to automatically identify the *H* and *V* Comps recovered by SOBI (Sun et al., 2021). DANS generates two discriminant indices (*DI_H_* and *DI_V_*) for every SOBI component to index temporal response selectivity. *DI_H_* and *DI_V_* were defined as the normalized difference between two signed amplitudes of saccade‐related potentials (SRPs) in response to long saccades in opposite directions (*DI_H_*: SRP_Right_–SRP_Left_; *DI_V_*: SRP_Up_–SRP_Down_) in proportion to the maximum difference value (maximum *DI* = 1.0). In this study, SRP refers to the event‐related potentials triggered by target onsets in dot tracking task. SRP amplitude was computed as the median amplitude within 200–1200 ms after target onset with a baseline correction window of 500 ms prior to target onset. DANS also generated two similarity indices (*SI_H_* and *SI_V_*) to index the resemblance of a component's scalp projection with the known prototypical scalp projection maps of *H* and *V* Comps. *SI_H_* and *SI_V_* were defined as the normalized correlation between the scalp projection of a SOBI component and the projection of a prototypical *H* or *V* Comps, respectively (normalization by the maximum value; maximum = 1.0). Examples of prototypical maps of *H* or *V* Comps can be found in previous work using BSS in ocular artifact removal (such as in Plochl et al., 2012; Vigário, 1997) or in Figure S1. If an *SI* is smaller than 0.5, then *SI* is set to 0, so that the corresponding component is effectively excluded from consideration because a component with *SI* < 50% of the *SI_MAX_* is unlikely to be the single best candidate capturing the horizontal or vertical saccade‐related component. Finally, horizontal and vertical scores (*H* and *V* scores), defined as the product of *DI* and *SI*, were computed for each component. The components with the largest nonzero *H* or *V* scores were the final selected *H* or *V* Comps, respectively. DANS selected *H* and *V* Comps for each participant are shown in Figure S1, whereas the grand averaged plot can refer to our previous study (Sun et al., 2021).

#### 2.4.3 Construction of individual‐specific linear regression models of gaze position using dot track data

The scatter diagrams of the relationship between horizontal, vertical SRP amplitudes (*Amp_SRP_H_*, *Amp_SRP_V_*) and target gaze positions (*X_t_*, *Y_t_*) for each participant are shown in Figure S2A (for scatter diagrams of *Amp_SRP_H_* vs. *X_t_*) and Figure S2B (for scatter diagrams of *Amp_SRP_V_* vs. *Y_t_*). It can be seen from the scatter diagrams that SRP amplitude and gaze position show a linear relationship in all participants. Therefore, linear regression analysis was used to fit the relationship. Target positions (*X_t_*, *Y_t_*) were defined as dependent variables, whereas SRP amplitudes (*Amp_SRP_H_*, *Amp_SRP_V_*) were defined as independent variables. The linear equations can be established as follows:

$$X_{t}\;=a_{x}\;\times Amp_{SRP\_H}+b_{x}\times Amp_{SRP\_V}+c_{x}$$

$$Y_{t}\;=a_{y}\;\times Amp_{SRP\_H}+b_{y}\times Amp_{SRP\_V}+c_{y}$$

Least‐square estimation was used to estimate the slope coefficients of independent variables ($a_{x},\;b_{x},\;a_{y},\;\mathrm{and}\;b_{y}$) and intercepts ($c_{x}\;\mathrm{and}\;c_{y}$), which will be applied in performance testing of the EEG‐VET.

In summary, the method of the EEG‐VET is composed of a SOBI method, a DANS algorithm, and two linear equations. SOBI algorithm was used to separate EEG signals into different components, whereas DANS algorithm was used to automatically identify *H* and *V* Comps from SOBI‐recovered components. The linear regression analysis was used to establish linear equations between ocular components (*Amp_SRP_H_*, *Amp_SRP_V_*) and gaze positions (*X_t_*, *Y_t_*). The performance of the EEG‐VET was evaluated by accuracy, precision in a saccadic eye movement, and RMSE between target and measured movement trajectory by EEG‐VET in a smooth pursuit task.

### 2.5 Model performance test using smooth pursuit task accuracy and precision

In evaluating the EEG‐VET, individual specific model constructed from dot tracking task data was used to (1) predict gaze positions during the left and right saccades that began the smooth pursuit trials; (2) predict gaze positions during the tracking of a moving target in the same smooth pursuit task. In measuring the errors in the two types of prediction, accuracy and precisions were computed for prediction (1) and a RMSE in tracking was computed for prediction (2).

Both accuracy and precision are defined in degrees of visual angle. Since both target and gaze positions are originally given as distances on the display screen, below we show derivation of the final visual angle measures from the distance measures. The accuracy in mm can be defined as

$$Accurancy\;=\sqrt{{\left(X_{t}-X_{m}\right)}^{2}+{\left(Y_{t}-Y_{m}\right)}^{2}}$$

In the equation, $X_{t}$ and $Y_{t}$ are the horizontal and vertical coordinates of a target location, whereas $X_{m}$ and $Y_{m}$ are the average horizontal and vertical coordinates of the measured gaze location by eye‐tracker when participants were required to fix their eyes on the target point.

The OnscreenDistance refers to the distance from a location on the screen to the center of the screen. Therefore, the OnscreenDistance of the target point is

$$\;OnScreenDistance_{T}=\sqrt{{\left(X_{t}-\frac{xscreen}{2}\right)}^{2}+{\left(Y_{t}-\frac{yscreen}{2}\right)}^{2}}\;$$

whereas the OnscreenDistance of average gaze location measured by eye‐tracker is

$$\;OnScreenDistance_{G}=\sqrt{{\left(X_{m}-\frac{xscreen}{2}\right)}^{2}+{\left(Y_{m}-\frac{yscreen}{2}\right)}^{2}}\;$$

The visual angle related to on‐screen distance can be calculated via

$$Angle\;=\tan^{-1}\;\left(\frac{OnScreenDistance}{Distance}\right)$$

xscreen,yscreenrefers to the width and height of the screen. Distancerefers to the distance from the screen to the participant's eye, which was 600 mm in this study. The accuracy in degrees of visual angle can be expressed as the deviation in degrees between the actual gaze direction and gaze direction measured by the eye‐tracker, with the point of origin determined by the position of the eye. It can be estimated via

$$\begin{array}{ccc} VisualAngleAcurrancy\; & = & \frac{180}{\pi }\times \cos^{-1}\;\left\{\frac{1}{2}\times \left[\frac{\cos Angle_{t}^{2}+\cos Angle_{m}^{2}}{\cos Angle_{t}\times \cos Angle_{m}}\right.\right.\\ & & \left.\left.-\frac{Accuracy^{2}\times \cos Angle_{t}\times \cos Angle_{m}}{Distance^{2}}\right]\right\} \end{array}$$

Precision (in degrees of visual angle) is defined as the ability of the eye‐tracker to reliably reproduce the same gaze point measurement. It is calculated via the root mean square from the *n* successive data points (in degrees of visual angle $\theta_{i}$) between gaze location measured by eye‐tracker ($X_{mi},\;Y_{mi}$) to ($X_{m(i+1)},\;Y_{m(i+1)}$)

$$\begin{array}{ccc} \;\theta_{i} & = & \frac{180}{\pi }\times \cos^{-1}\;\left\{\frac{1}{2}\times \left[\frac{\cos Angle_{mi}^{2}+\cos Angle_{m\left(i+1\right)}^{2}}{\cos Angle_{mi}\times \cos Angle_{m\left(i+1\right)}}\right.\right.\\ & & \left.\left.-\frac{OnScreenDistance_{mi,\;m\left(i+1\right)}^{2}\times \cos Angle_{mi}\times \cos Angle_{m\left(i+1\right)}}{Distance^{2}}\right]\right\} \end{array}$$

The $OnScreenDistance_{mi,\;m(i+1)}$ is the distance between ($X_{mi},\;Y_{mi}$) and ($X_{m(i+1)},\;Y_{m(i+1)}$). Then the precision in degrees of visual angle can be estimated via

$$VisualAnglePrecision\;=\sqrt{\frac{1}{n}\sum_{i=1}^{n}\theta_{i}^{2}}$$

The accuracy and precision of the EEG‐VET in individuals were estimated using 64 trials of saccade eye movements starting from the screen center (252, 142.5 mm) to a left point (229, 142.5 mm) or a right point (275, 142.5 mm) with 32 trials for each.

Meanwhile, we also calculated the accuracy and precision across all participants, which were estimated using the dot tracking tasks across all participants, including 576 trials of the saccade eye movements (16 participants with 32 trials for each) starting from the screen center (252, 142.5 mm) to each target location (see Figure 1b). It is used to compare the performance between EEG‐VET and SMI REDn eye‐tracker.

### 2.6 Model performance: Errors in tracking a moving target measured by RMSE

RMSE between target trajectory and estimated eye movement trajectory in the smooth pursuit task by EEG‐VET was computed for each pursuit trial according to the following equation where i represents time sampling point, total sampling point = 180:

$$RMSE\;=\sqrt{\sum_{i=1}^{total\;sampling\;point}\frac{{\left(predicted\;coordinate\;-\;Target\;coordinate_{i}\right)}^{2}}{total\;sampling\;point}}$$

Two‐way repeated measures analysis of variance (ANOVA) with four speeds (1, 5, 9, and 19°/s) and two directions (left and right) as within factors was used to test the effects of speeds (1, 5, 9, and 19°/s), directions (left vs. right) and the interaction effect. Analyses were conducted using SPSS (Version 22, IBM) with a significance level of .05.

## 3 RESULTS

### 3.1 Model construction: Goodness of fit of the individual‐specific linear repression model in mapping SRP amplitude to gaze position

For each participant, two linear equations with both *H* and *V* Comps as independent variables and gaze position *X* or *Y* as dependent variables were established reasonable. The correlation coefficients (*r*) and their respective *p* values for the linear regression analysis are summarized in Table 1. The correlation coefficient between SRPs (*Amp_SRP_H,_ Amp_SRP_V_*) and *X_t_* is .95 ± .04, which accounts for over 80% (R square) of variance in horizontal gaze coordinates. whereas *Y_t_* is .84 ± .13 which accounts for only over 60% of variance in vertical gaze coordinates. Note also that a majority of the *p*‐values are below .01. The relative inferior fits in the vertical dimension is expected in part because in the current work we did optimize on the removal of blinks and minimizing the correlations between eye lids opening and closing during the blinks and during the up and down eye movement. This is to be further considered in the next stage of the EEG‐VET development.

**FIGURE 3:**
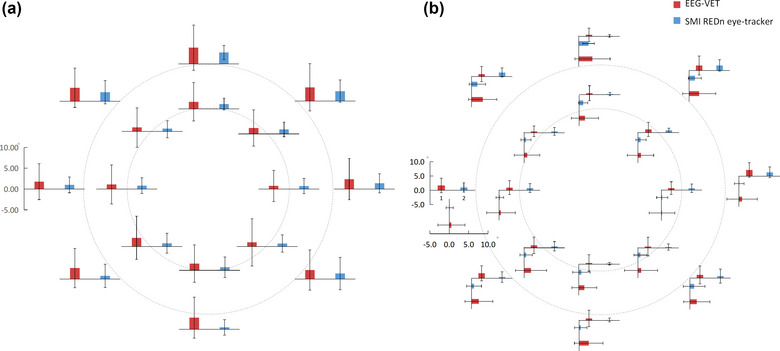
Comparison of accuracy/precision between electroencephalogram‐based virtual eye‐tracker (EEG‐VET) (red bar) and SMI REDn eye‐tracker (blue bar) on 16 target points: (a) the accuracy/precision is combined from the *X* and *Y* axes as a general description; (b) the accuracy/precision is separated along *X* and *Y* axes. The bars indicate the accuracy value, whereas the error lines indicate the precision value. *The average accuracy of EEG‐VET across all participants and all target points is 2.05° Å} 0.93°, whereas the precision is 4.76° Å}0.55° of visual angle. For SMIREDn eye‐tracker, the accuracy is 1.19° Å} 0.67°,whereas the precision is 2.23° Å} 0.61° of visual angle. This difference is significant (accuracy: t(15) = 5.66, p < .01; precision: t(15) = 13.97, p < .01). The accuracy is better when the target points are in the inner ring than outer ring both the EEG‐VET and the commerical eye tracker (EEG‐VET: t(7) = 6.23, p < .01; eye tracker: t(7) = 2.71, p = .03)*.

To give a concrete impression of the model goodness in the visual space, we show the group measures of accuracy and precision for each of the 16 target positions in dot tracking task (Fig. 3) contrasting the performances from the EEG‐VET (FIg. 3a) and the commericial eye tracker used in the present study (Fig. 3b). *We that as these accuracy/precision data were calculated across all participants, we do not recommend comparing these with the above or other accuracy/precision results calculated on single participant separately*. While the performance of the current method appeared not as good as the eye tracker, they are on the same order of magnitude. Further optimization beyond the present proof of concept is planned to further significantly improve the EEG‐VET performance.

**FIGURE 4:**
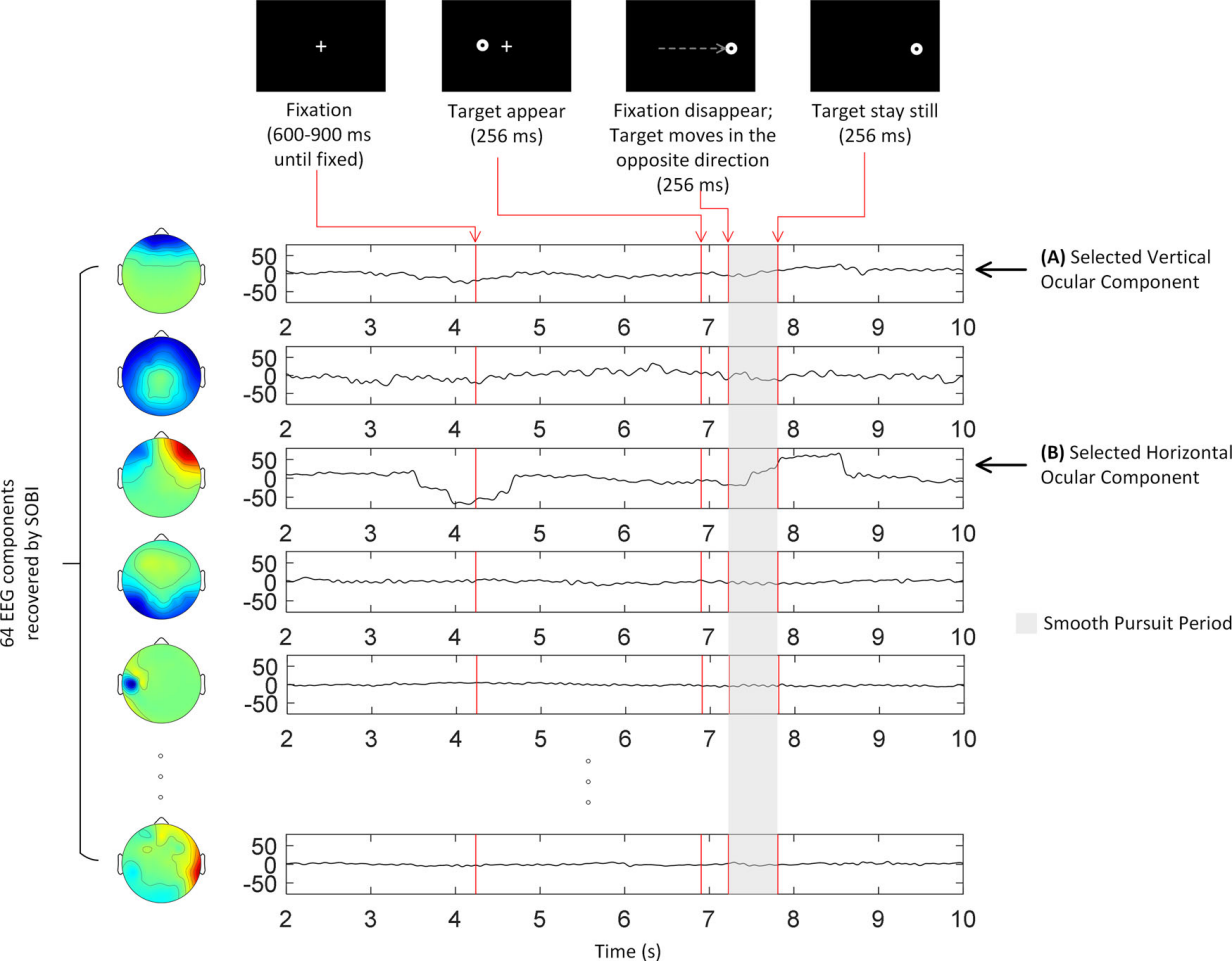
An example illustrating a selective contribution of the Horizontal ocular component to tracking of a moving target while the other neuronal components (line 2,4,5) are insertive to horizontal eye movement. Gray block: smooth pursuit period.

Although the accuracy of commercial eye‐tracker systems is often reported by manufacturers to be <0.5° of visual angle, the true gaze point of remote eye‐trackers is often found to be worse than 1° of visual angle, even in controlled environments (Blignaut et al., 2014; Nyström et al., 2013). Besides, it can be seen from Figure 4 that the accuracy/precision of EEG‐VET is below twice of SMI REDn eye‐tracker in most of the target points. Therefore, the accuracy of this first attempt EEG‐VET approaches that of commercial eye‐trackers.

**TABLE 1:** Goodness of the individual‐specific linear models for using saccade‐related potential (*Amp_SRP_H_*, *Amp_SRP_V_*) to predict target gaze position (*X_t_*, *Y_t_*) for each participant's data from the dot tracking task.

	Using both *Amp_SRP_H_* and *Amp_SRP_V_* to predict *X_t_* (mm)			Using both *Amp_SRP_H_* and *Amp_SRP_V_* to predict *Y_t_* (mm)		
#P	*r*	*p* for SRP_H	*p* for SRP_V	*r*	*p* for SRP_H	*p* for SRP_V
P1	.91	<.01**	.21	.84	<.01**	<.01**
P2	.96	<.01**	.12	.60	.01*	<.01**
P3	.96	<.01**	.80	.94	.99	<.01**
P4	.96	<.01**	.16	.89	<.01**	<.01**
P5	.98	<.01**	<.01**	.92	<.01**	<.01**
P6	.93	<.01**	.65	.91	<.01**	<.01**
P7	.96	<.01**	.13	.88	<.01**	<.01**
P8	.99	<.01**	.65	.96	<.01**	<.01**
P9	.97	<.01**	<.01**	.91	.75	<.01**
P10	.96	<.01**	.02*	.76	.06	<.01**
P11	.92	<.01**	.16	.90	.55	<.01**
P12	.98	<.01**	<.01**	.86	.52	<.01**
P13	.94	<.01**	.72	.73	.01*	<.01**
P14	.97	<.01**	.27	.47	.45	.01*
P15	.95	<.01**	.05	.94	.15	<.01**
P16	.99	<.01**	.16	.83	.68	<.01**
P17	.85	<.01**	.34	.90	.81	<.01**
P18	.86	<.01**	.19	.94	.04*	<.01**
Mean	.95			.84		
SD	.04			.13		

* stands for p < 0.05 while ** for p < 0.01.

Abbreviations: #P, identifier number of participant; *p*, significant value; *r*, correlation coefficient; SRP_H, horizontal saccade–related potential; SRP_V, vertical saccade–related potential.

### 3.2 Performance of EEG‐VET in tracking gaze positions in the smooth pursuit task

**FIGURE 5:**
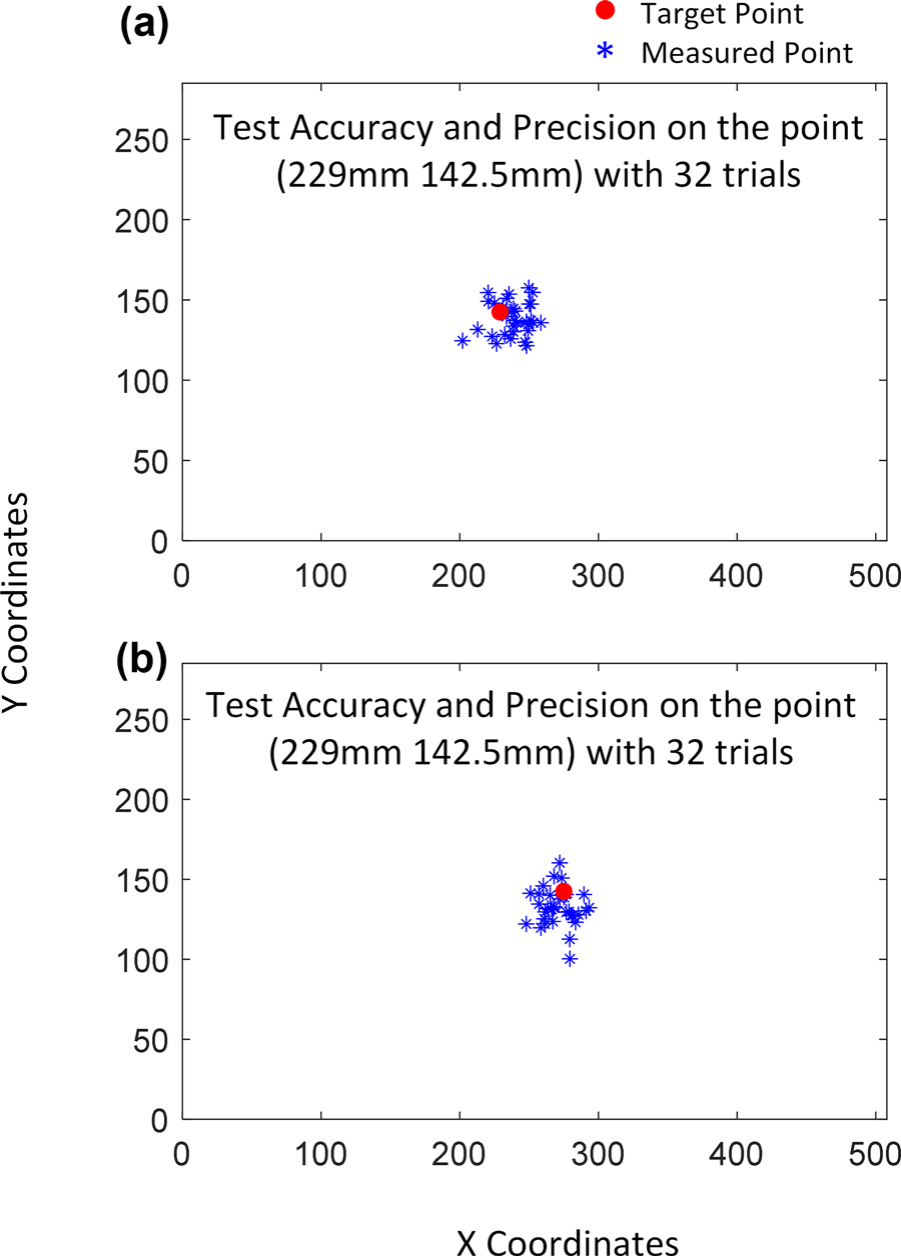
Illustration of model performance in estimating the gaze positions after the saccades to the left and right target position during the smooth pursuit task (Fig. 1d, the 3rd screen). (a) demonstration of a left target point (red dot at [229, 142.5 mm]) and 32 predicted gaze points by EEG‐VET (blue asterisk points); (b) demonstration of a right target point (275, 142.5 mm) and 32 predicted gaze points by EEG‐VET (blue asterisk points) for one participant *with an accuracy of 0.920° of visual angle and a precision of 1.510° of visual angle*.

Using the models fitted with data from the first dot tracking task, we estimated gaze positions during the subsequent task of smooth pursuit. While we have previously shown that DANS selected H and V components were highly selective for horizontal and vertical movement in the dot tracking task (Sun et al., 2021), here we also show such selectivity in the smooth pursuit task (see an example of a single pursuit trial in one participant in Fig. 4). For this example participant, the estimated gaze positions after the initial horizontal saccadic eye movement to the left and right of the fixation point are shown in Fig. 5a and 5b respectively. Figure 3 illustrates the performance of the EEG‐VET in one participant with Figure 2a and 2b showing predicted gaze positions (Blue) to the Left and Right target positions (Red) respectively in the smooth pursuit task. Across all 18 participants, the accuracy across all participants was 1.008° ± 0.357° of visual angle, whereas the precision was 2.347° ± 0.580° of visual angle.

To give a concrete impression of the model goodness in the visual space, we show the group measures of accuracy and precision for each of the 16 target positions in dot tracking task (Fig. 3) contrasting the performances from the EEG‐VET (FIg. 3a) and the commericial eye tracker used in the present study (Fig. 3b). While the performance of the current method appeared not as good as the eye tracker, they are on the same order of magnitude. Further optimization beyond the present proof of concept is planned to further significantly improve the EEG‐VET performance.

**FIGURE 6:**
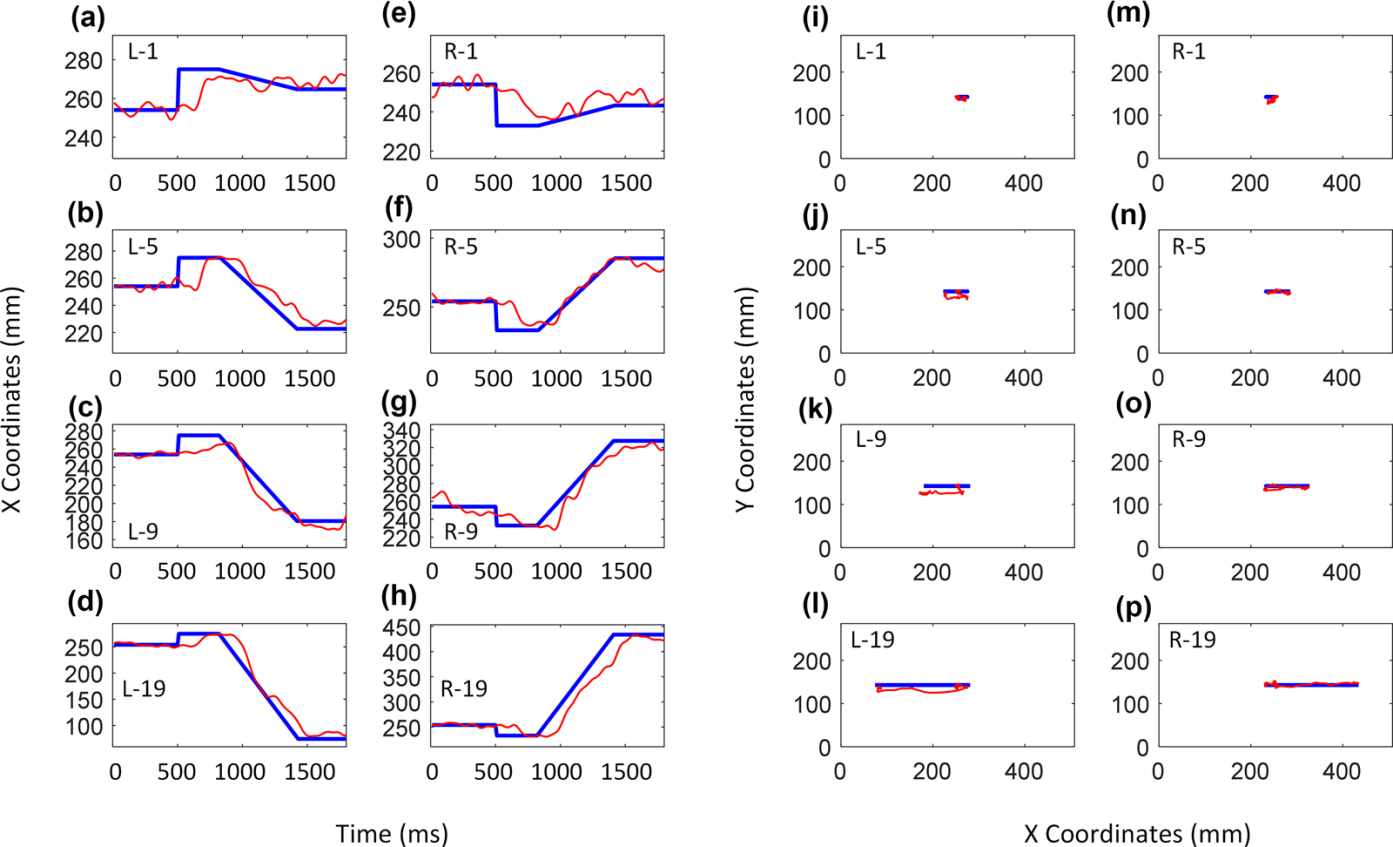
Demonstration of target *X* (a–h, blue line), *X* predicted by electroencephalogram‐based virtual eye‐tracker (EEG‐VET) (a–h, red line), target gaze trajectory (i–p, blue line), and gaze trajectory predicted by EEG‐VET (I–P, red line) in one participant performing a smooth pursuit task at different pursuit speeds (1, 5, 9, and 19°/s) and directions (left and right) with labels as L‐1, L‐5, L‐9, L‐19, R‐1, R‐5, R‐9, R‐19. Data are from the same participant as in Fig. 5. For dynamic display, see Supplementary video.

**FIGURE 7:**
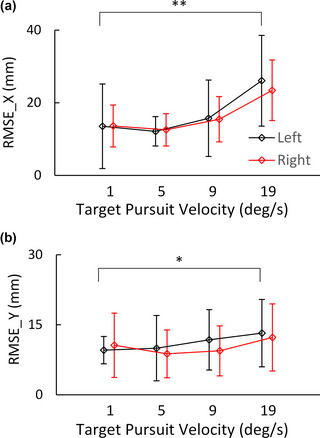
Performance of EEG‐VET in estimating gaze position during the tracking of a moving target in the smooth pursuit task. (a) prediction error in the horizontal coordinate and (b) prediction error in vertical coordinate. * and ** indicate significant Speed effects on estimation error. Error bars are standard deviations (N=18).

In evaluating EEG‐VET performance during the tracking of a moving target, we first show the performance of the EEG‐VET, in a single participant, for tracking the gaze position with four different speeds (1, 5, 9, and 19°/s) and two different directions (left and right) in the same participant shown in Fig. 5. Figure 6a–h illustrates the actual target's *X* coordinate *X* and the estimated gaze position *X* predicted by EEG‐VET as a function of time, whereas Figure 6i–p shows the actual change in target trajectory and the estimated trajectory measured by EEG‐VET across time. The blue line indicates the actual target gaze trajectory, whereas the red line indicates the gaze trajectory predicted by EEG‐VET. The estimated waveforms across four speeds and two directions follow the target waveform fairly well indicating that the EEG‐VET perform fairly well. The group performance is shown in Fig. 7.

### 3.3 Performance of EEG‐VET with changes in direction and speed of eye movement

Two‐way repeated measure ANOVA was performed on the RMSE of the EEG‐VET with Direction and Speed as within factors. Tracking speed had a significant effect on *X* RMSE (*F*(3, 51) = 29.408; *p* < .001; partial η^2^ = .634) and *Y* RMSE (*F*(3, 51) = 4.010; *p* = .012; partial η^2^ = .191). There was no significant effect of direction on the RMSE *X* (*F*(1, 17) = .215; *p* = .648; partial η^2^ = .013) and RMSE *Y* observed (*F*(1, 17) = .612; *p* = .445; partial $\eta^{2}\;$= .035), which indicates no difference in the performance of EEG‐VET between pursuing left and right. No significant Speed by Direction interaction effects were found. It is important to point out that given the nature of the task, the actual gaze position during the pursuit would be falling behind the target movement. Thus the error in the horizontal dimension in part contains this distance lag. This in part explains why onver average Y RMSE appeared apparently smaller than RMSE.

## 4 DISCUSSION

### 4.1 Concept of an EEG‐based virtual eye tracker

Building upon previous work (Tang, Sutherland, Mckinney, et al., 2006, Sun et al., 2019, Sun et al., 2021), in the context of horizontal eye movement, the present study demonstrated that it is possible to build individualized predictive models of gaze positions from EEG without the aid of an eye‐tracker. The results show that it is possible to obtain an accuracy as high as 0.920° of visual angle and precision of 1.510° of visual angle in one participant that is comparable to the accuracy and precision of electrooculogram (EOG)‐based eye tracking (Young & Sheena, 1975) and combined EOG–EEG based eye‐tracking systems (Joyce et al., 2002). The prediction errors of EEG‐VET across all participants are greater than those of a commercialized eye‐tracker but no more than twice of it. Such a “resolution” is sufficient for many types of experimental studies and applications.

Ocular artifacts associated with eye movement in EEG present a major problem in cognitive neuroscience and clinical research. Instead of minimizing artifacts by restricting eye movement and removing artifacts from the EEG data as documented in a number of review papers (Croft & Barry, 2000; Islam et al., 2016; Jiang et al., 2019; Mannan et al., 2018; Urigüen & Garcia‐Zapirain, 2015), the present study presents a novel approach to this problem by transforming the artifacts into informative signals for predicting gaze positions. Specifically, the EEG‐VET method requires a directed saccadic eye movement task, to generate the EEG data to “calibrate” the EEG‐VET for predicting gaze positions in subsequent tasks involving eye movement. This work offers a first prototype of the EEG‐VET for predicting gaze positions from EEG data alone in horizontal smooth pursuit tasks.

### 4.2 The usage and advantage of the EEG‐VET

The method of the EEG‐VET is similar to that of commercial eye‐trackers as both require data collected during a sequence of directed saccadic eye movement to known positions in order to build the model for estimating gaze positions and then the EEG‐VET can be used for tracking gaze position not only during directed eye movement but also during any subsequent tasks within the same EEG session, including free eye movement. The EEG‐VET can potentially provide many advantages over widely used commercial eye‐trackers. Most significant is that the use of the EEG‐VET facilitates the tracking of eye movement during EEG studies without the need for any additional hardware. Beyond predicting eye movement, this system can provide ocular artifact‐free EEG signal by summing non‐ocular components, which is especially suitable for Brain Computer Interface applications as it provides eye movement information and ocular contamination‐free EEG signal simultaneously. Finally, because the system relies on information recovered from internal signals (i.e., EEG), it can be used to measure eye movements in contexts where the use of an eye‐tracker would prove impractical or impossible such as during sleep or free viewing without the limitation of a screen.

For the computation time, the calculation speed of SOBI largely depends on the speed of the computing device, the number of EEG channels, the sampling rate, and the recording time length. For using the dot tracking task as the calibration task (calibration is normally used in most of commercialize eye‐trackers). We have done a preliminary exploration using data from a single participant and found that it is possible to achieve asymptotic performance within 4 repetitions of the dot positions (Sun et al., 2020). A general recommendation would only be available after further studies with more participants. For the readers’ reference, the calibration task used in the present study took around 2–3 min in this study. Using SOBI to deriving the H and V components from the 64‐channel EEG collected during 3 min dot tracking task. The computation time needed by DANS algorithm and linear regression analysis is negligible. Therefore, the time required for model parameter estimation is no more than a few minutes. After model establishment, the computing of gaze position is negligible.

### 4.3 Limitations and future work

As a newly emerging method, the current performance of EEG‐VET is not yet as good as the commercially available eye trackers especially in the vertical direction. The performance has been evaluated in a narrow context of horizontal smooth pursuit task. Future work may fall into four categories. The first is to further improve the efficiency and effectiveness of the entire EEG‐VET process, starting from the design, evaluation criterion, and the optimal length of the calibration task to using of potentially even better algorithms. The second is to expand the work beyond tracking of moving target in the horizontal directions by recognizing and considering the unique properties of vertical versus horizontal eye movements in EEG‐VET design. The third is to remove the constraint of head movement by introducing innovations of the EEG‐VET take allow the computation to take head direction information into consideration. The fourth is to expand the performance evaluation into a more diverse range of application scenarios.

In summary, we hope that the potential technical capability afforded by this new concept of VET will inspire new areas of investigation, such as neurophysiological investigation of natural reading, learning and memory during sleep, better devices for neural feedback control, and other clinical neuroscience problems.

It is interesting to note that the recovered *H* Comps were more informative than *V* Comps. *H* Comps had higher correlation coefficients with *X* gaze position (*r* = .95 ± .04) than *V* Comps with *Y* gaze position (*r* = .84 ± .13). We suspect that the signals due to eye blinks may be mixed with the signals due to vertical eye movement for looking up and down can naturally involve the opening and closing of the eye lids. Because the *V* Comp is one of the independent variables in the linear regression model, the final model performance measures reported here should be considered conservative. Better performance can potentially be expected if blinks and vertical eye movements could be dissociated in the behavioral task of dot tracking.

## 5 Conclusion

In conclusion, we offered a proof of a concept that the use of a novel EEG‐VET can enable the simultaneous collection of EEG and eye movement data without the need for additional hardware. This work is the first to utilize the information contained in typically discarded ocular artifact components in order to track eye movement from EEG signal alone. The current version of EEG‐VET can achieve levels of accuracy and precision that almost approach those of commercial eye‐trackers.

## AUTHOR CONTRIBUTIONS

**Rui Sun**: Conceptualization; formal analysis; methodology; software; validation; writing—original draft; writing—review & editing. **Andy S. K. Cheng**: Performance testing; writing—review & editing. **Cynthia Chan**: Behavioral and electrophysiology data curation; writing—original draft; writing—review & editing. **Janet Hsiao**: Conceptualization; data curation; funding acquisition; investigation; behavioral and electrophysiology methods; project administration; resources; supervision; writing original draft; writing—review & editing. **Adam John Privitera**: Statistical analysis; Writing—review & editing. **Junling Gao**: Writing—review & editing. **Ching‐hang Fong**: Coding; Writing—review & editing. **Ruoxi Ding**: Writing—review & editing. **Akaysha Tang**: Conceptualization; formal analysis; funding acquisition; investigation; methodology; project administration; resources, software; supervision; validation; visualization; writing—original draft; writing—review & editing.

## CONFLICT OF INTEREST STATEMENT

All authors declare no conflicts of interest.

### PEER REVIEW

The peer review history for this article is available at https://publons.com/publon/10.1002/brb3.3205.

## Supporting information

Figure S1 The identified *H* and *V* Comps from all components separated by SOBI for each participant (P1–P18).
Figure S2 Model goodness: the correlations between saccadic eye movement target position (*X_t_*, *Y_t_*) and *H* and *V* Comps’ SRPs amplitude (*Amp_SRP_H_*, *Amp_SRP_V_*), shown for all 18 participants.

## Data Availability

The data that support the findings of this study are available from the corresponding author upon reasonable request.
