# Supplementary material for: Tracking gaze position from EEG: Exploring the possibility of an EEG‐based virtual eye‐tracker
Source: Brain Behav. 2023 Sep 18;13(10):e3205. doi: 10.1002/brb3.3205 (PMC10570499; doi:10.1002/brb3.3205)
Supplement: Supplementary file 1 — Figure S1 The identified H and V Comps from all components separated by SOBI for each participant (P1–P18). Figure S2 Model goodness: the correlations between saccadic eye movement target position (Xt , Yt ) and H and V Comps’ SRPs amplitude (AmpSRP_H , AmpSRP_V ), shown for all 18 participants. [file BRB3-13-e3205-s002.docx]

**Supplementary Material**

Tracking Gaze Position from EEG: Exploring the Possibility of an EEG-based Virtual Eye-Tracker

Rui Sun^1,3^, Andy Cheng^1^, Cynthia Chan^2^, Janet Hsiao^2^, Adam John Privitera^4^, Junling Gao^5^, Ching-hang Fong^1^, Ruoxi Ding^6^, Akaysha C. Tang^3,7**^

*1. Department of Rehabilitation Sciences, the Hong Kong Polytechnic University*

*2. Department of Psychology, the University of Hong Kong*

*3. The Laboratory of Neuroscience for Education, the University of Hong Kong*

*4. Centre for Research and Development in Learning, Nanyang Technological University*

*5. Centre of Buddhism Studies, the University of Hong Kong*

*6. China Center for Health Development Studies, Peking University*

*7. Neural Dialogue, Shenzhen, China*

*
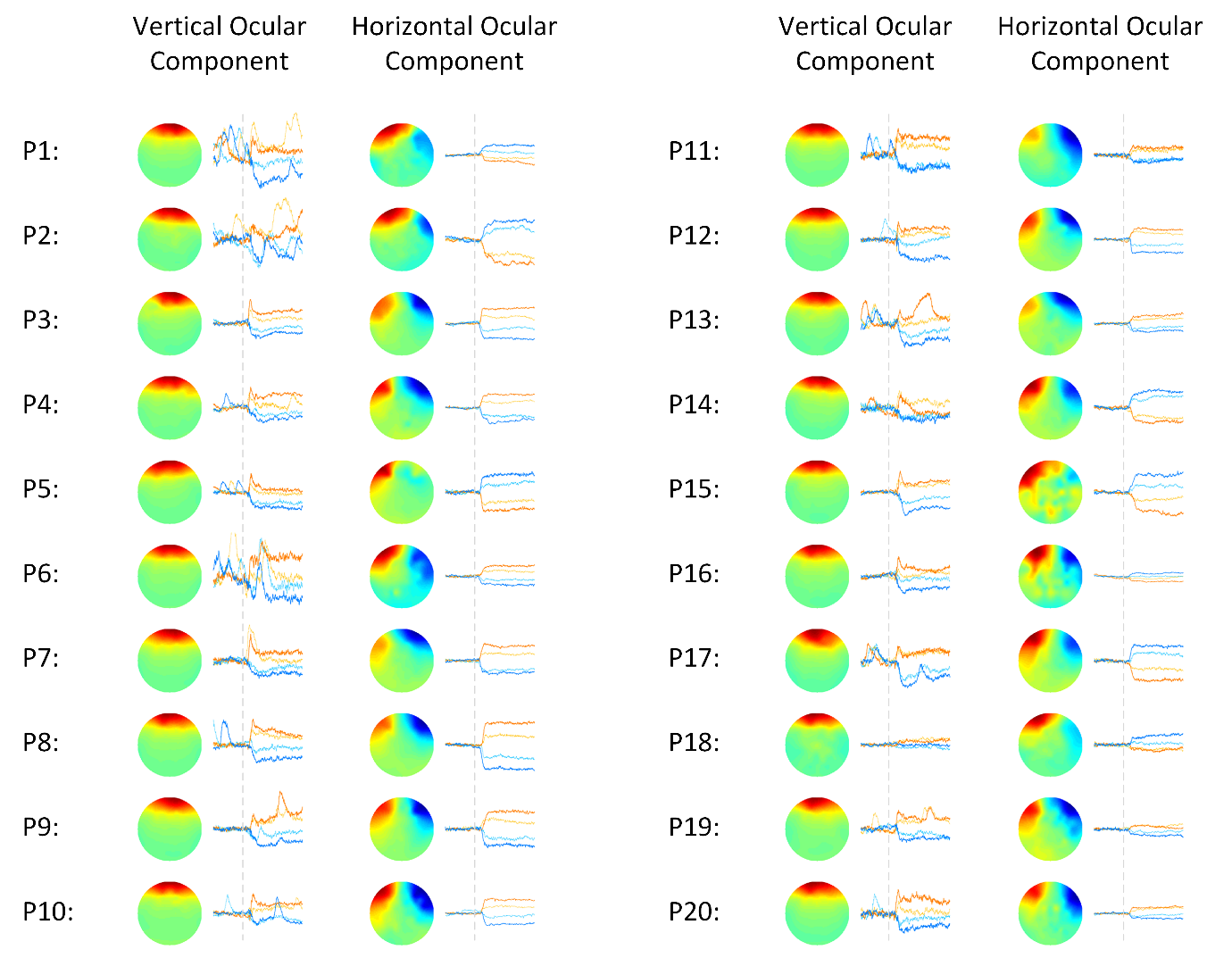
*

**Supplementary Fig. 1**. The identified H and V Comps from all components separated by SOBI for each participant (P1 – P18). The left topographies are the scalp projections of each H or V Component while the right curves are the saccade related potentials (SRPs) of each H or V Component. The Grey dashed lines mark the time point of target dot appears (stimulus onset).

*
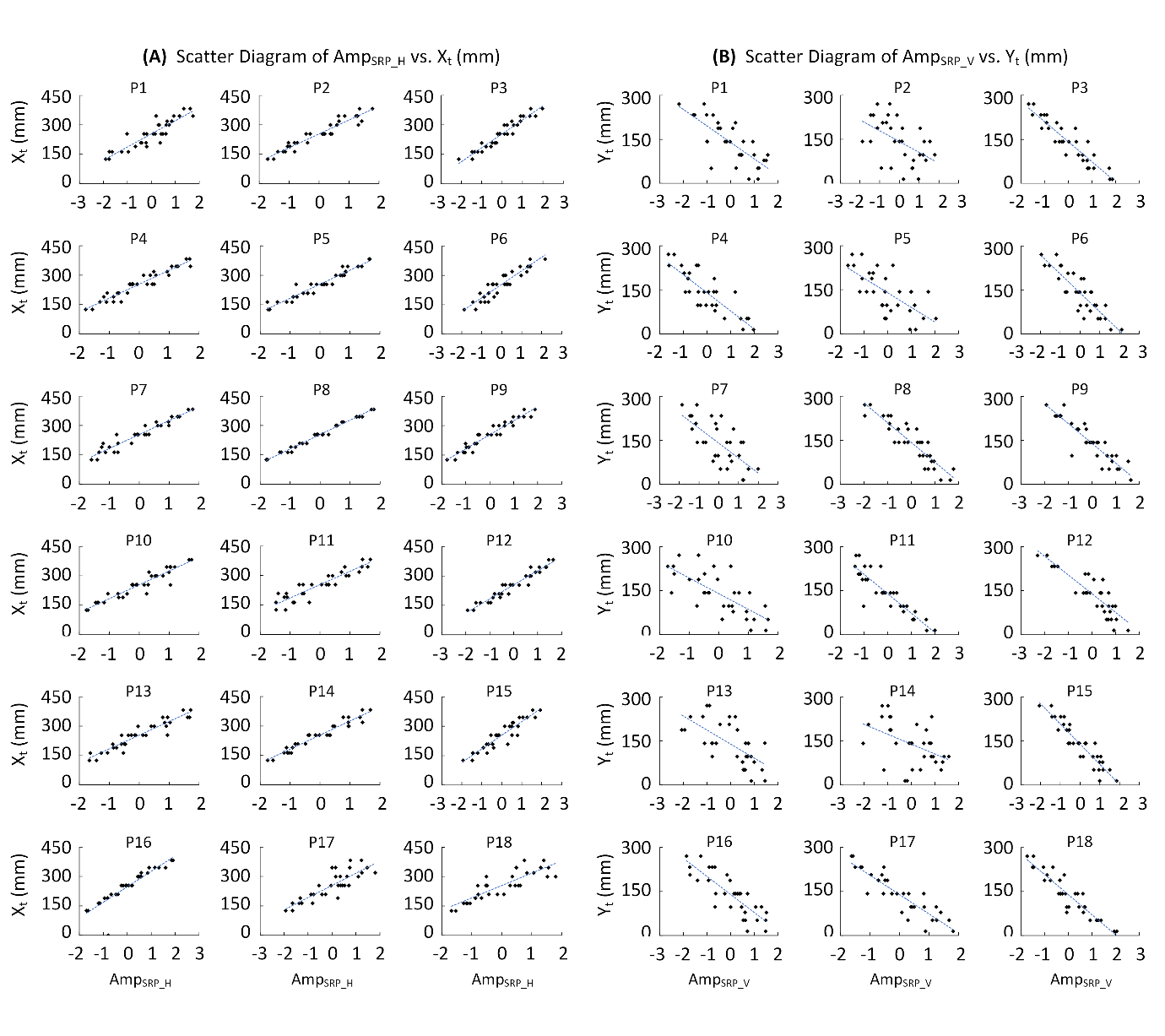
*

**Supplementary Fig. 2** Model goodness: the correlations between saccadic eye movement target position (X_t_, Y_t_) and H and V comps’ SRPs amplitude (Amp*_SRP_H_*, Amp*_SRP_V_*), shown for all 18 participants. **(A)** scatter diagrams of Amp*_SRP_H_* vs. X_t_. **(B)** scatter diagrams of Amp*_SRP_V_* vs. Y_t_. The blue line: linear regression fitting.
